# Association between time to surgery and hospital mortality in patients with community-acquired limb necrotizing fasciitis: an 11-year multicenter retrospective cohort analysis

**DOI:** 10.1186/s12879-024-09501-y

**Published:** 2024-06-23

**Authors:** Chi Ho Lau, Lowell Ling, Jack Zhenhe Zhang, Pauline Yeung Ng, Cheuk Yan Chan, Alwin Wai Tak Yeung, Ka Man Fong, Jacky Ka Hing Chan, Gary Ka Fai Au, Ting Liong, Manimala Dharmangadan, Fu Loi Chow, Koon Ngai Lam, Kai Man Chan, Steven Ling, Anna Lee

**Affiliations:** 1 Department of Anaesthesia and Intensive Care, Prince of Wales Hospital, Shatin, Hong Kong SAR China; 2 Department of Anaesthesia and Intensive Care, The Chinese University of Hong Kong, Prince of Wales Hospital, Shatin, Hong Kong SAR China; 3 Critical Care Medicine Unit, Li Ka Shing Faculty of Medicine, The University of Hong Kong, Pok Fu Lam, Hong Kong SAR China; 4 Department of Adult Intensive Care, Queen Mary Hospital, Pok Fu Lam, Hong Kong SAR China; 5 Department of Intensive Care, Pamela Youde Nethersole Eastern Hospital, Chai Wan, Hong Kong SAR China; 6 Department of Medicine & Geriatrics, Ruttonjee and Tang Shiu Kin Hospitals, Wan Chai, Hong Kong SAR China; 7 Department of Intensive Care, Queen Elizabeth Hospital, Yau Ma Tei, Hong Kong SAR China; 8 Department of Medicine, Tseung Kwan O Hospital, Tseung Kwan O, Hong Kong SAR China; 9 Department of Intensive Care, Kwong Wah Hospital, Yau Ma Tei, Hong Kong SAR China; 10 Department of Intensive Care, United Christian Hospital, Kwun Tong, Hong Kong SAR China; 11 Department of Intensive Care, Princess Margaret Hospital, Kwai Chung, Hong Kong SAR China; 12 Department of Intensive Care, Yan Chai Hospital, Tsuen Wan, Hong Kong SAR China; 13 Department of Intensive Care Unit, Department of Medicine & Geriatrics, Caritas Medical Centre, Sham Shui Po, Hong Kong SAR China; 14 Department of Intensive Care, North District Hospital, Sheung Shui, Hong Kong SAR China; 15 Intensive Care Unit, Department of Medicine, Alice Ho Miu Ling Nethersole Hospital, Tai Po, Hong Kong SAR China; 16 Department of Intensive Care, Tuen Mun Hospital, Tuen Mun, Hong Kong SAR China

**Keywords:** Risk factors, Debridement, Soft tissue infections, Anti-bacterial agents, Electronic health records

## Abstract

**Background:**

Necrotizing fasciitis (NF) is a rare but potentially life-threatening soft tissue infection. The objective of this study was to assess the association between timely surgery within 6 h and hospital mortality in patients with limb NF, and to describe the trends in patients with NF, time to surgery and standardized mortality ratio (SMR) over 11 years.

**Methods:**

This was a multicenter, retrospective cohort study of all intensive care unit patients who had emergency surgery within 24 h of hospitalization for limb NF between April 1, 2008 and March 31, 2019 in Hong Kong. Timely surgery was defined as the first surgical treatment within 6 h of initial hospitalization. Appropriate antibiotics were achieved if the patient was given antibiotic(s) for all documented pathogens prior to or on day of culture results. The primary outcome was hospital mortality.

**Results:**

There were 495 patients (median age 62 years, 349 (70.5%) males) with limb NF treated by surgery within 24 h of hospitalization over the 11 years. Appropriate antibiotic(s) were used in 392 (79.2%) patients. There were 181 (36.5%) deaths. Timely surgery was not associated with hospital mortality (Relative Risk 0.89, 95% CI: 0.73 to 1.07) but admission year, advanced age, higher severity of illness, comorbidities, renal replacement therapy, vasopressor use, and type of surgery were significant predictors in the multivariable model. There was an upward trend in NF diagnosis (1.9 cases/year, 95% CI: 0.7 to 3.1; *P* < 0.01; R^2^ = 0.60) but there was no downward trend in median time to surgery (-0.2 h/year, 95% CI: -0.4 to 0.1; *P* = 0.16) or SMR (-0.02/year, 95% CI: -0.06 to 0.01; *P* = 0.22; R^2^ = 0.16).

**Conclusions:**

Among patients operated within 24 h, very early surgery within 6–12 h was not associated with survival. Increasing limb NF cases were reported each year but mortality remained high despite a high rate of appropriate antibiotic use and timely surgical intervention.

**Supplementary Information:**

The online version contains supplementary material available at 10.1186/s12879-024-09501-y.

## Introduction

**Fig. 1:**
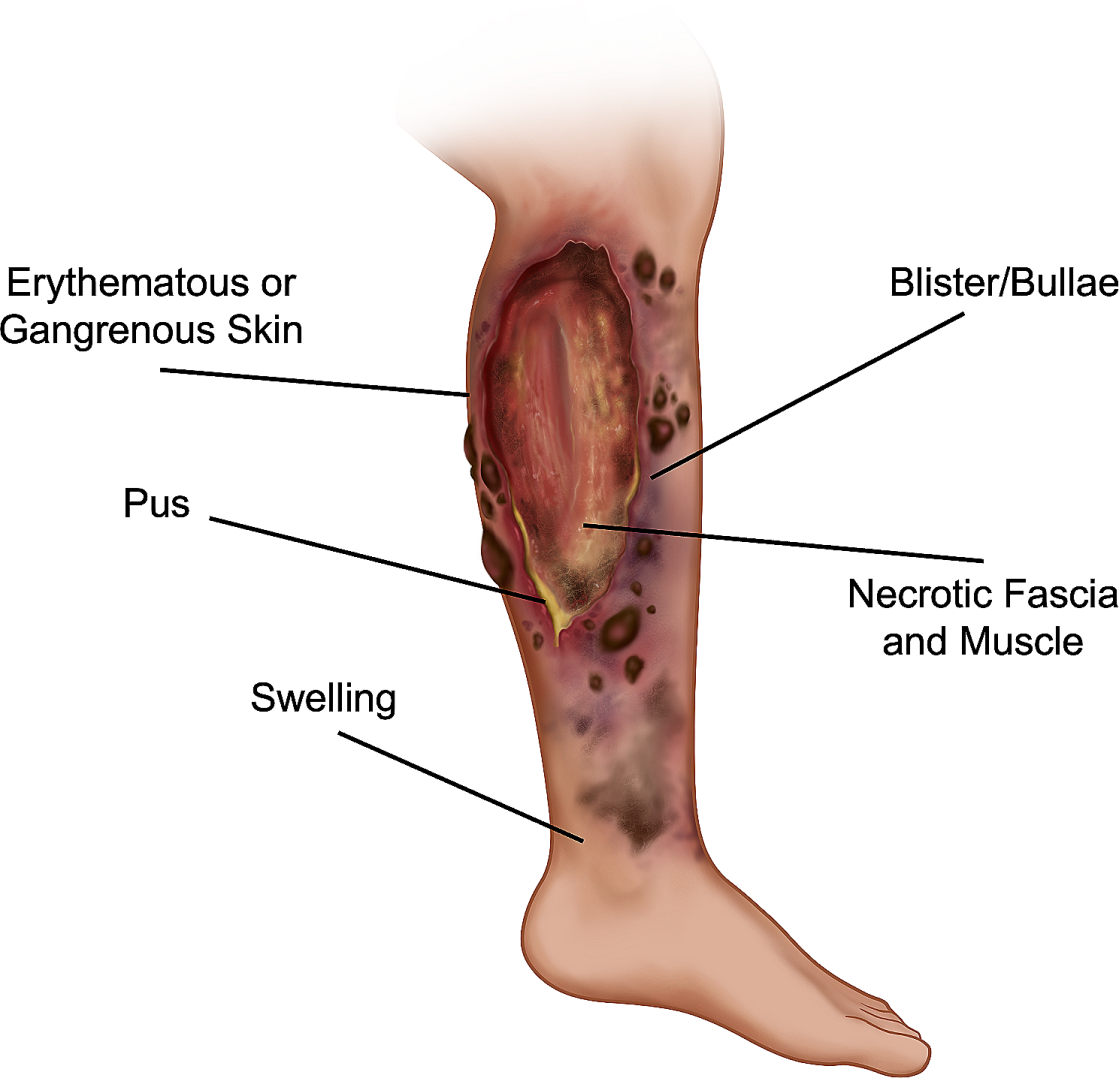
Leg with necrotizing fasciitis

Necrotizing fasciitis (NF) is a rare but life-threatening severe soft tissue infection involving the skin, subcutaneous tissue, and superficial fascia (Fig. 1) [1, 2]. The annual incidence of NF ranges from 1.7 cases per 100,000 population in New Zealand [3] to 11.6 cases per 100,000 in Taiwan [4]. NF is categorized into polymicrobial type I and monomicrobial type II infections [1]. Type I NF is usually caused by a combination of aerobic and anaerobic organisms in patients with comorbidities [5]. In contrast, Type 2 NF affects healthy patients and is often due to group A *Streptococcus* (GAS) or *Staphylococcus aureus* [6]. Environmental factors also determine causative pathogens as Methicillin-resistant *Staphylococcus aureus* (MRSA) is a substantial cause of NF in communities with a high prevalence of MRSA [7]. In tropical and coastal regions, *Vibrio* and *Aeromonas* species are common causes of NF due to the temperate climate and seawater exposure [8].

A recent meta-analysis found that the mortality of NF remains high at 21% and survival has not improved over the last 20 years [9]. This may be partly due to the difficulty in diagnosis [10]. Presentation is highly variable, ranging from disproportionate pain relative to skin erythema, blisters, or gangrenous changes of skin [10]. Once the diagnosis is suspected, the key management priorities include prompt surgery with aggressive debridement of necrotic tissue for histological diagnosis and source control, as well as timely administration of appropriate broad-spectrum antibiotics [11]. Additional clindamycin is sometimes advocated for synergistic toxin suppression for GAS NF [12].

Conclusive high-quality evidence on the optimal medical and surgical interventions for NF are lacking [13]. Factors associated with increased mortality in NF include high severity of illness by APACHE score [14], presence of bacteraemia [15], hypotension [16], advanced age [17] and comorbidities [18]. More importantly, reducing the time to surgical treatment within 6 to 12 h may be a modifiable risk factor to improve survival [9, 11, 19–21]. However, many of these studies did not evaluate whether appropriate antibiotics were given [9, 20–22]. To address this knowledge gap, the primary objective of this study was to assess the association between time to surgery within 6 h and hospital mortality in critically ill patients with community-acquired limb NF. The secondary objective was to describe the trend in limb NF cases, time to surgery and standardized mortality ratio (SMR) between 2008 and 2018. We hypothesised that surgical treatment within 6 h would be an independent factor associated with improved survival after adjustment for other covariates. Furthermore, time to surgery has decreased and standardized mortality ratio has improved over the last decade.

## Methods

### Study design and population

This was a multicenter, retrospective cohort study conducted at 15 public hospitals in Hong Kong. This study was approved by The Joint Chinese University of Hong Kong – New Territories East Cluster Clinical Research Ethics Committee (2020.078), The Hong Kong East Cluster Research Ethics Committee (HKECREC-2020-030), The Institutional Review Board of The University of Hong Kong/Hospital Authority Hong Kong West Cluster (UW20-288), The Research Ethics Committee of Kowloon Central/Kowloon East (KC/KE-20-007/ER-1), The Kowloon West Cluster Research Ethics Committee (KW/EX-20-077(148-01)) and New Territories West Cluster Research Ethics Committee (NTWC/REC/20,058). Each individual ethics committee granted waiver of informed consent for this study. The STROBE guideline was used to ensure proper reporting of methods, results, and discussion [23].

All adult intensive care unit (ICU) patients with a diagnosis of NF who had emergency surgical treatment between 1 April 2008 and 31 March 2019 (inclusive) were included. For patients with recurrent ICU admissions during the same hospitalization, only data from their first ICU admissions was used. Exclusion criteria included: necrotizing fasciitis primarily involving sites other than the limbs (head and neck, chest, back, abdomen, perineum or groin areas) and missing hospital discharge status. Patients who only had emergency surgery after 24 h of hospital admission were excluded. The rationale was to focus on patients who presented with community acquired NF.

### Data collection and variable definitions

Patients fulfilling the inclusion and exclusion criteria were identified using Clinical Data Analysis and Reporting System (CDARS), an electronic health database of the Hospital Authority in Hong Kong. CDARS contains inpatient and outpatient clinical information including clinical notes, laboratory results, microbiological results, operation records, diagnosis, procedures of inpatient, outpatient and Accident and Emergency Department care. We identified patients with a diagnosis of NF by hospital discharge International Classification of Diseases, Ninth Revision, Clinical Modification (ICD-9-CM) code of Necrotizing fasciitis (728.86:0). The Charlson Comorbidity Index Score [24] was calculated using all inpatient and outpatient ICD-9-CM codes collected for each patient within 10 years before hospital admission [18]. We collected the Acute Physiology and Chronic Health Evaluation (APACHE) IV score [25], use of organ function supportive therapies including vasopressors, mechanical ventilation and renal replacement therapy (RRT), antibiotics use, and microbiology results from cultures of body fluids, wound swabs or surgical specimens.

Time to surgery was defined as the time interval between hospital admission and the start of emergency surgery. Timely surgical treatment was defined as within six hours (or within 12 h in the sensitivity analysis) between the time of initial hospital admission and emergency surgery. Surgery within these thresholds were recently shown in a meta-analysis to be associated with improved survival [9]. The primary outcome was hospital mortality which is defined as all-cause mortality at time of hospital discharge.

Type of surgery was classified into three categories:

(1) debridement only, in which only surgical debridement or other equivalent procedures were performed throughout the entire hospital admission;

(2) amputation first, in which amputation was performed within the first surgical session documented during the hospital admission; and (3) debridement followed by amputation, in which only debridement or other equivalent procedures were performed during the first surgical session, followed by amputation of any affected limb(s) in subsequent sessions.

Microbiological results were classified into monomicrobial, polymicrobial or culture-negative infections, according to the culture results of body fluids, wound swabs or surgical specimens obtained within 48 h of hospital admission. Appropriate antibiotics were achieved if the patient was given antibiotic(s) for all documented pathogens prior to or on day of culture results. Operation booking category was defined as the priority category of the emergency surgery which was either urgent (within 1 h) or semi-urgent (within 24 h) at time of surgery booking. This was used to provide further stratification of severity prior to surgery as patients with higher severity of disease are usually prioritized urgently for surgery.

### Data analysis

Demographics and other baseline characteristics were expressed as descriptive statistics. Continuous data were reported as mean and standard deviation (SD) or median and interquartile range (IQR) as appropriate after checking visually and using Shapiro-Wilk’s test for normality. Frequency (%) was reported for categorical variables. The APACHE IV adjusted standardized mortality ratio was calculated by the number of deaths divided by the expected number of deaths during the 11 years.

Complete case analysis was used. We performed Chi-square tests to examine the association between categorical variables and mortality. Mann-Whitney U tests were used to assess the differences in the duration of ICU and hospital stays between time-to-surgery groups. We adjusted for age groups, APACHE IV groups, Charlson Comorbidity Index Score, type of infection, presence of bacteraemia, use of appropriate antibiotics, mechanical ventilation, RRT, vasopressor, type of surgery and operation booking category as confounders and year of admission in a multivariable model after constructing a directed acyclic graph (DAG) [26] using DAGitty software (http://www.dagitty.net/). In the DAG model (Supplemental Fig. 1), we were interested in the direct effect of time to surgery on the risk of hospital mortality. A modified Poisson regression model with a robust error variance [27] was used to assess the association between timely surgery (< 6 h) and hospital mortality. The relative risk (RR) of hospital mortality with the associated 95% confidence interval (95% CI) were reported. The model discrimination performance was assessed using an area under the receiver operating characteristic curve (AUROC) and a calibration belt was drawn to visually compare the observed versus expected risk of hospital mortality [28]. A sensitivity analysis was performed to assess the association between timely surgery within 12 h and hospital mortality. We used a quantile regression to estimate the trend in median time to surgery over 11 years and a linear regression to assess the hospital mortality trend over this study period. The statistical analyses were performed using Stata version 18.0 (StataCorp, College Station, TX). The level of significance was set at *p* < 0.05.

## Results

**Fig. 2:**
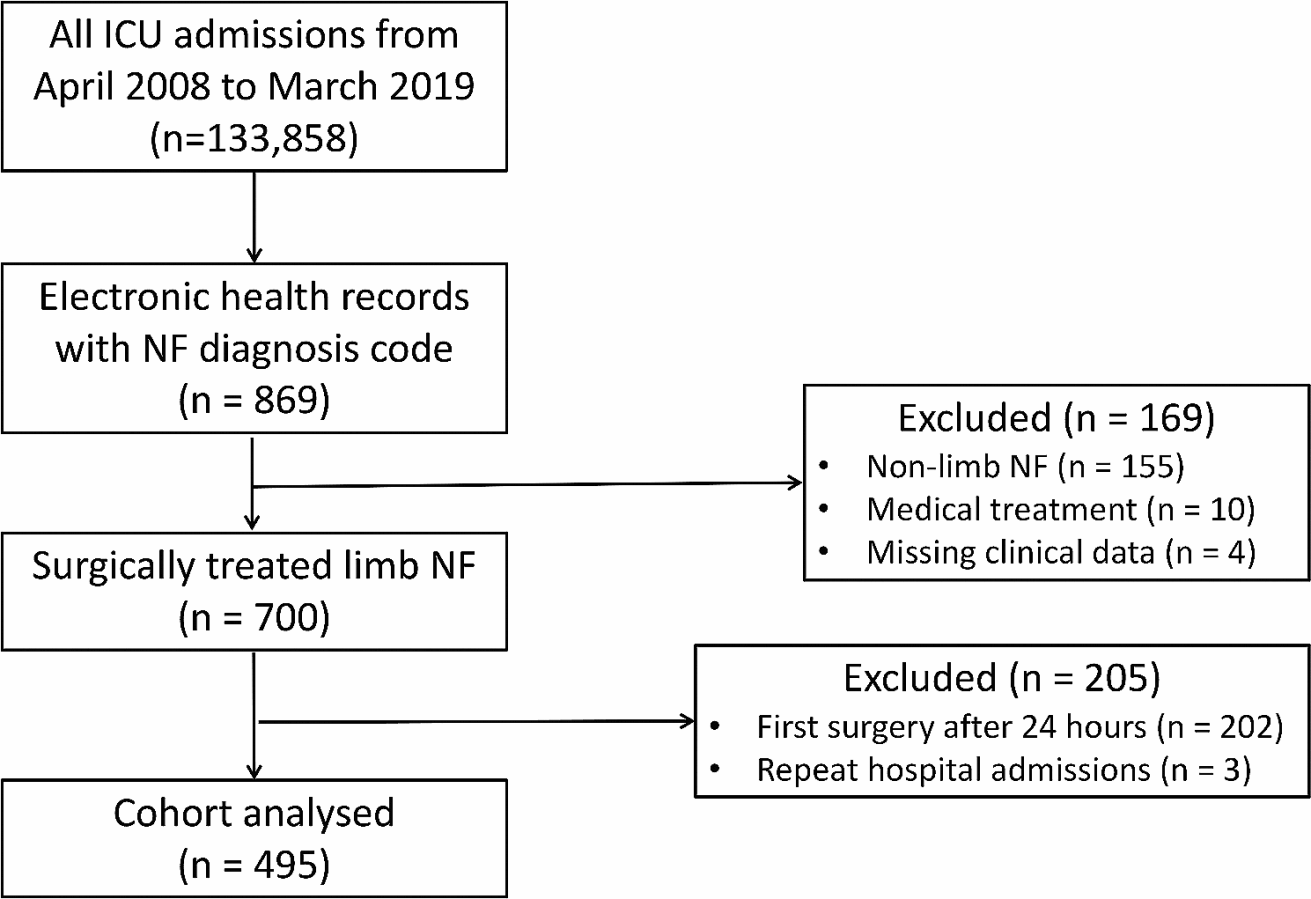
Patient flow diagram

During the 11-year study period between 1 April 2008 and 31 March 2019, there were 133,858 admissions to the 15 general ICUs in Hong Kong. Of these 133,858 ICU admissions, 869 critically ill patients had NF and 700 received surgical treatment (Fig. 2). Among these 700 NF cases, 495 had surgical treatment within 24 h of hospitalization and were included in the final study cohort (Fig. 2, Supplemental Fig. 2). The number of surgeries performed within 6 h, 6 to 12 h and 12 to 24 h were 212 (42.8%), 153 (30.9%) and 130 (26.3%) respectively.

The patient demographics and clinical characteristics are shown in Table 1 and Supplemental Table 1. Most patients (71%) had monomicrobial infections. The most common organisms (Table 2) for patients with monomicrobial infections were *Streptococcus pyogenes* (34%) and *Vibrio* species (22.4%). Amongst the whole cohort, the mortality rates for patients with *Streptococcus pyogenes* and *Vibrio* species were 24.4% (30/123) and 39.1% (34/87), respectively. Quinolones (67%), penicillins (62%) and clindamycin (56%) were the most frequently used antimicrobial agents (Table 3). At least 12% of patients were not given appropriate antibiotics (Table 1, Supplemental Table 2). The antimicrobial resistance rates for various organisms are shown in Supplemental Table 3. Among the 170 patients with bacteraemia, a total of 91 (53.5%) patients had the same pathogen isolated from wounds.

**Table 1:** Patient demographics and clinical characteristics

Characteristics	Dead (*n* = 181)	Alive (*n* = 314)	Total (*n* = 495)
Median (IQR) age, years	66 (56–78)	60 (50–71)	62 (52–74)
Males, n (%)	122 (67.4)	227 (72.3)	349 (70.5)
Median (IQR) APACHE IV	117 (94–144)	72 (56–87)	83.0 (64–109)
Comorbidities, n (%)			
Diabetes mellitus	49 (27.1)	52 (16.6)	101 (20.4)
Cardiovascular	65 (35.9)	58 (18.5)	123 (24.8)
Malignancy	28 (15.5)	21 (6.7)	49 (9.9)
Liver	61 (33.7)	37 (11.8)	98 (19.8)
Renal	38 (21.0)	15 (4.8)	53 (10.7)
Rheumatological	8 (4.4)	3 (1.0)	11 (2.2)
Chronic pulmonary diseases	17 (9.4)	19 (6.1)	36 (7.3)
HIV/AIDS	4 (2.2)	12 (3.8)	16 (3.2)
Median (IQR) Charlson Comorbidity Score	2 (0–4)	0 (0–2)	0 (0–2)
Type of infection, n (%)			
Monomicrobial	140 (77.3)	213 (67.8)	353 (71.3)
Polymicrobial	32 (17.7)	68 (21.7)	100 (20.2)
No growth	9 (5.0)	33 (10.5)	42 (8.5)
Bacteraemia, n (%)	96 (53.0)	74 (23.6)	170 (34.3)
Operation booking category, n (%)			
Semi-urgent	24 (13.3)	59 (18.8)	83 (16.8)
Urgent	150 (82.9)	250 (79.6)	400 (80.8)
Unknown	7 (3.9)	5 (1.6)	12 (2.4)
Median (IQR) time to surgery, hours	7.8 (4.1–13.1)	6.7 (3.7–11.8)	7.2 (3.9–12.4)
Type of surgery, n (%)			
Debridement only	75 (41.4)	202 (64.3)	277 (56.0)
Amputation only	73 (40.3)	51 (16.2)	124 (25.1)
Debridement then amputation	33 (18.2)	61 (19.4)	94 (19.0)
Median (IQR) number of operations	2 (1–3)	4 (2–5)	3 (2–5)
Appropriate antibiotic(s)*, n (%)			
Appropriate	148 (81.8)	244 (77.7)	392 (79.2)
Inappropriate	24 (13.3)	37 (11.8)	61 (12.3)
Unknown	9 (5.0)	33 (10.5)	42 (8.5)
Mechanical Ventilation, n (%)	178 (98.3)	262 (83.4)	440 (88.9)
Vasopressor, n (%)	92 (50.8)	116 (36.9)	208 (42.0)
Renal replacement therapy, n (%)	88 (48.6)	38 (12.1)	126 (25.5)
Median (IQR) ICU length of stay, days	3.4 (1.3–10.1)	4.7 (2.9–7.8)	4.4 (2.4–8.4)
Median (IQR) hospital length of stay, days	5.0 (1.6–20.3)	36.5 (22.7–61.7)	27.1 (11.0-46.7)
ICU mortality, n (%)	134 (74.0)	0 (0.0)	134 (27.1)

*Appropriate antibiotics was defined as use of antibiotics that covers the sensitivity pattern of confirmed pathogens within 24 h of the microbiological result

AIDS, acquired immune deficiency syndrome; APACHE IV, Acute Physiology And Chronic Health Evaluation IV; HIV, human immunodeficiency virus; ICU, intensive care unit; IQR, interquartile range

**Table 2:** Type of microorganisms

**Microbiology in patients with monomicrobial NF**	
**Pathogen**	*n* = 353 (%)
**Gram positive organisms**	
*Streptococcus Pyogenes*	120 (34.0)
Other *Streptococcus* (*S. agalactiae, S. Anginosus*, Group C or Group G)	42 (11.9)
Methicillin-sensitive *Staphylococcus aureus* (MSSA)	25 (7.1)
Methicillin-resistant *Staphylococcus aureus* (MRSA)	5 (1.4)
Coagulase negative *Staphylococci species*	18 (5.1)
**Gram negative organisms**	
*Aeromonas*	19 (5.4)
*Vibrio*	79 (22.4)
*Enterobacteriaceae*	28 (7.9)
*Pseudomonas*	9 (2.5)
Other Gram negatives	5 (1.4)
**Anaerobes**	3 (0.8)
**Microbiology in patients with polymicrobial NF**	
**Pathogen**	*n* = 100 (%)
**Gram positive organisms**	
*Streptococcus Pyogenes*	3 (3.0)
Other *Streptococcus* (*S. agalactiae, S. Anginosus*, Group C or Group G)	15 (15.0)
Methicillin-sensitive *Staphylococcus aureus* (MSSA)	6 (6.0)
Methicillin-resistant *Staphylococcus aureus* (MRSA)	6 (6.0)
Coagulase negative *Staphylococci species*	10 (10.0)
Gram Positive Bacilli (*Enterococcus, Micrococcus)*	11 (11.0)
**Gram negative organisms**	
*Aeromonas*	2 (2.0)
*Vibrio*	8 (8.0)
*Enterobacteriaceae*	8 (8.0)
*Pseudomonas*	4 (4.0)
Other Gram negatives	9 (9.0)
**Anaerobes**	9 (9.0)
***Mycobacteria***	1 (1.0)
***Yeast/Candida***	8 (8.0)

**Table 3:** Antimicrobial use on Day 1 of hospitalization

Antimicrobial class	Number of patients *n* = 495 (%)
Penicillin *(benzylpenicillin, ampicillin, amoxicillin-clavulanate, cloxacillin)*	308 (62.2)
Antipseudomonal penicillin *(piperacillin-tazobactam, piperacillin)*	198 (40.0)
Cephalosporin *(cefazolin, cefotaxime, cefuroxime, ceftriaxone, ceftazidime, cefepime)*	111 (22.4)
Quinolones *(ciprofloxacin, levofloxacin)*	332 (67.1)
Aminoglycoside *(amikacin, gentamicin)*	49 (9.9)
Macrolide *(clarithromycin)*	8 (1.6)
Carbapenem *(meropenem, imipenem-cilastatin)*	103 (20.8)
Tetracycline *(doxycycline, minocycline)*	9 (1.8)
Vancomycin	87 (17.6)
Linezolid	97 (19.6)
Clindamycin	279 (56.4)
Metronidazole	67 (13.5)
Cefoperazone-sulbactam	14 (2.8)
Colistin	1 (0.2)

Of the 495 patients, 212 (42.8%) had surgery within six hours. Timely surgery within six hours of initial hospital admission was not associated with hospital mortality in both unadjusted (RR 0.86, 95% CI: 0.63 to 1.17) and adjusted analysis (RR 0.89, 95% CI: 0.73 to 1.07) (Table 4). In the multivariable analysis, the significant factors associated with hospital mortality were the year of admission, higher age groups, higher severity of illness groups, comorbidity score, RRT, vasopressor use and type of surgery (Table 4). The model had excellent discrimination (AUROC 0.92, 95% CI: 0.90 to 0.95) and good calibration performance (*p* = 0.32; Supplemental Fig. 3). Three hundred and sixty-three (73.3%) patients had surgery within 12 h. In the sensitivity multivariable analysis, there was no association between timely surgery within 12 h of initial hospital admission and hospital mortality (Supplemental Table 4); however, the same factors in Table 4 were associated with hospital mortality. The sensitivity analysis model also had excellent discrimination (AUROC 0.92, 95% CI: 0.90 to 0.95) and good calibration performance (*p* = 0.46; Supplemental Fig. 4).

**Table 4:** Association between time to surgery (< 6 h) and risk of hospital mortality (unadjusted and adjusted relative risk) after taking hospital intensive care unit clustering effect into account in the model

Factors	Mortality, *n* (%)	Relative Risk (95% CI)	*p* value	Adjusted Relative Risk (95% CI)	*p* value
Time to Surgery (h)					
≥6	110 (38.9)	1.00		1.00	
<6	71 (33.5)	0.86 (0.63 to 1.17)	0.34	0.89 (0.73 to 1.07)	0.21
Year of admission					
2008	20 (55.6)	1.00		1.00	
2009	14 (45.2)	0.81 (0.47 to 1.41)		0.74 (0.49 to 1.11)	
2010	20 (50.0)	0.90 (0.61 to 1.33)		0.74 (0.48 to 1.16)	
2011	17 (36.2)	0.65 (0.38 to 1.12)		0.61 (0.44 to 0.86)	
2012	16 (32.0)	0.58 (0.32 to 1.04)		0.63 (0.44 to 0.89)	
2013	16 (39.0)	0.70 (0.37 to 1.33)	< 0.001	0.53 (0.37 to 0.77)	< 0.001
2014	14 (35.0)	0.63 (0.38 to 1.05)		0.63 (0.41 to 0.96)	
2015	6 (13.0)	0.23 (0.13 to 0.44)		0.34 (0.25 to 0.45)	
2016	22 (37.3)	0.67 (0.40 to 1.12)		0.63 (0.41 to 0.98)	
2017	19 (35.2)	0.63 (0.42 to 0.96)		0.71 (0.49 to 1.02)	
2018	17 (33.3)	0.60 (0.42 to 0.86)		0.58 (0.38 to 0.89)	
Age groups (years)					
<45	8 (11.9)	1.00		1.00	
45–64	71 (34.5)	2.89 (1.59 to 5.25)		1.93 (1.23 to 3.03)	
65–74	46 (43.0)	3.60 (1.86 to 6.99)	< 0.001	2.22 (1.32 to 3.73)	< 0.01
≥75	56 (48.7)	4.08 (2.00 to 8.32)		2.55 (1.50 to 4.32)	
APACHE IV groups					
<60	5 (4.9)	1.00		1.00	
60–89	32 (17.8)	3.63 (2.06 to 6.37)		2.27 (1.28 to 4.01)	
90–119	58 (50.0)	10.2 (5.52 to 18.84)	< 0.001	4.82 (2.59 to 8.96)	< 0.001
≥120	86 (88.7)	18.09 (11.41 to 28.67)		8.04 (4.98 to 12.99)	
Charlson Comorbidity					
0	63 (23.1)	1.00		1.00	
1	15 (39.5)	1.71 (1.03 to 2.83)		1.30 (0.93 to 1.83)	
2	34 (44.7)	1.94 (1.36 to 2.76)	< 0.001	1.38 (1.11 to 1.73)	< 0.001
≥3	69 (63.9)	2.77 (2.28 to 3.36)		1.69 (1.46 to 1.95)	
Type of infection					
Monomicrobial	140 (39.8)	1.00		1.00	
Polymicrobial	32 (32.0)	0.81 (0.68 to 0.96)	0.01	0.97 (0.78 to 1.22)	0.39
No growth	9 (20.9)	0.54 (0.30 to 0.97)		0.69 (0.40 to 1.18)	
Bacteraemia					
No	85 (26.2)	1.00		1.00	
Yes	96 (56.5)	2.16 (1.76 to 2.65)	< 0.001	1.12 (0.89 to 1.40)	0.34
Inappropriate antibiotics					
No or unknown	157 (36.2)	1.00		1.00	
Yes	24 (39.3)	1.09 (0.76 to 1.55)	0.64	1.08 (0.79 to 1.46)	0.64
Mechanical ventilation					
No	3 (5.5)	1.00		1.00	
Yes	178 (40.5)	7.42 (2.97 to 18.53)	< 0.001	2.65 (0.91 to 7.73)	0.07
Renal replacement therapy					
No	93 (25.2)	1.00		1.00	
Yes	88 (69.8)	2.77 (2.15 to 3.57)	< 0.001	1.32 (1.10 to 1.60)	< 0.01
Vasopressor					
No	89 (31.0)	1.00		1.00	
Yes	92 (44.2)	1.43 (1.17 to 1.73)	< 0.01	1.25 (1.01 to 1.56)	0.04
Type of surgery					
Debridement only	75 (27.1)	1.00		1.00	
Amputation only	73 (58.9)	2.17 (1.77 to 2.67)	< 0.001	1.16 (1.00 to 1.33)	< 0.001
Debridement then amputation	33 (35.1)	1.30 (1.00 to 1.69)		0.87 (0.66 to 1.16)	
Urgency of surgery					
Semi-urgent	24 (28.9)	1.00		1.00	
Urgent	150 (37.5)	1.30 (0.94 to 1.79)	< 0.01	1.00 (0.74 to 1.35)	1.00
Unknown	7 (58.3)	2.02 (1.33 to 3.06)		0.99 (0.68 to 1.45)	

APACHE IV, Acute Physiology And Chronic Health Evaluation IV

**Fig. 3:**
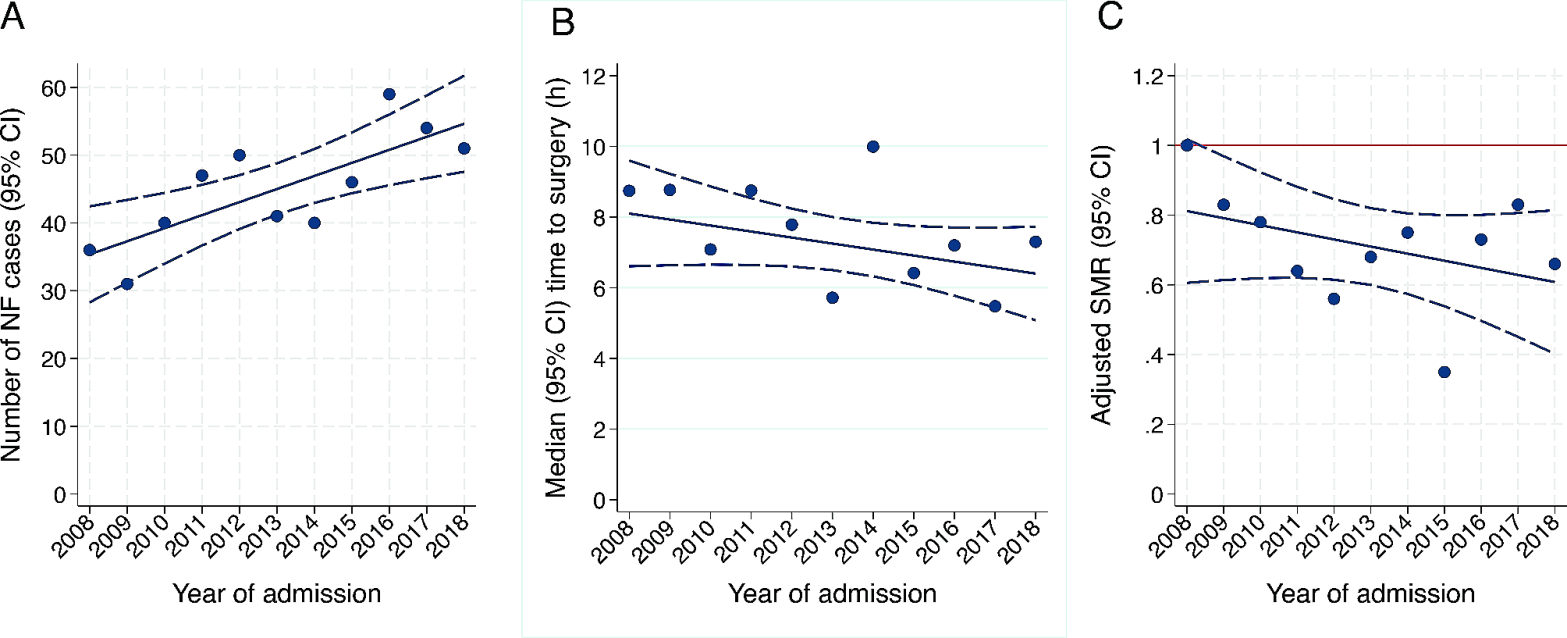
Trend over April 2008 to March 2019. (**A**) number of necrotizing fasciitis (NF), (**B**) median time (hours) to surgery and (**C**) severity of illness adjusted standardized mortality ratio (SMR)

In the unadjusted analyses, there was an increasing trend in the annual rate of NF cases over time (1.9 cases/year, 95% CI: 0.7 to 3.1; *p* < 0.01; R^2^ = 0.60; Fig. 3A). Although the median (IQR) time to surgery (hours) decreased from 8.1 (95% CI: 6.6 to 9.6) in 2008 to 6.4 (95% CI: 5.1 to 7.7) in 2018, the downward trend over time was not significant (-0.2 h/year, 95% CI: -0.4 to 0.1; *p* = 0.16; Fig. 3B). The ICU mortality was 27.1% (95% CI: 23.2–31.2%). The hospital mortality was 36.6% (95% CI: 32.3–41.0%). The APACHE IV adjusted SMR decreased from 0.81 (95% CI: 0.61 to 1.02) in 2008 to 0.61 (95% CI: 0.40 to 0.81) in 2018 although the downward trend over time was not significant (-0.02/year, 95% CI: -0.06 to 0.01; *p* = 0.22; R^2^ = 0.16; Fig. 3C).

Patients treated with surgery within six hours of initial hospital admission were not associated with a shorter length of stay in ICU (days) compared to those with a delayed surgery (median (IQR) 4.2 (2.5 to 7.8) versus 4.5 (2.4 to 8.8) days; *p* = 0.73). Likewise, surgery within six hours was not associated with a shorter duration of hospital stay (days) than those with a delayed surgery (median (IQR) 26.9 (10.7 to 46.6) versus 27.2 (11.0 to 46.8) days; *p* = 0.87).

## Discussion

The objective of this study was to use a large retrospective cohort of patients with community acquired limb NF to assess the association between time to surgery within 6 h and hospital mortality. We found that time to surgery within six hours (or 12 h) of hospital admission was not associated with hospital mortality after adjusting for multiple confounders. The overall APACHE IV adjusted SMR is low for critically ill patients with limb NF in Hong Kong, but there was no significant decline in either median time to surgery or SMR over the 2008 to 2018 period. Overall, there were approximately two additional cases of limb NF per year occurring over the study period. *Streptococcus pyogenes* was the most prevalent microorganism isolated (34%), whilst *Vibrio* species (22%) was also common.

Our baseline demographics and comorbidity profile were similar to previous studies [11, 17, 18]. In the current study, the median age was 62 years, with 71% being males; diabetes and cardiovascular disease (myocardial infarction, congestive heart failure, peripheral vascular disease, cerebrovascular disease) remained the most frequently reported comorbidities. Mild chronic liver disease was also prevalent (15%) which may be attributed to the high rate of chronic hepatitis carriage in our population. Patients with uncontrolled diabetes were at a higher risk of mortality than those with controlled diabetes [29]; however, the current dataset did not include the HbA1c level to examine this aspect. Nevertheless, it is important to highlight the SMR of limb NF patients in Hong Kong is low. Although this study did not demonstrate statistically significant improvement of SMR over time, it has been shown that the trend is consistent with the decreasing SMR of all ICU patients in Hong Kong during the same period [30]. Although the overall 37% hospital mortality rate reported is higher than average mortality rate of 20% reported in a recent meta-analysis which included all severity of patients [9], it is comparable to cohorts of critically ill patients with NF from France [31] and Netherlands [32].

Consistent with previous studies [2, 11], *S. pyogenes* was the most common microorganism isolated (34%); other beta-hemolytic streptococci, such as group C or G *Streptococcus* (12%), are also emerging [33]. Of note, our high rate of clindamycin resistance is within the reported clindamycin resistance rate ranging from 15% in the USA [34] to 96% in China [35]. Our cohort showed that *Vibrio* species (22%) was the second most common causative organism, reinforcing the need to consider empirical quinolone coverage during presentation. A systematic review showed that more than 95% of the *Vibrio vulnificus* NF cases occurred in the subtropical western Pacific and Atlantic coastal regions in the Northern Hemisphere [8]. However, climate change has been suggested as a cause of rising Vibrio infections in North America [36]. *Staphylococcus aureus*, both sensitive and resistant strains, were also quite prevalent in our cohort, consistent with a previous Taiwanese study [37].

Many studies have explored the predictors of adverse outcomes with this potentially fatal disease. Although there is no consensus for specific predictors of mortality so far, commonly agreed factors include advanced age, high severity of illness, delayed presentation and delayed intervention, and positive blood culture [38, 39]. Our study also showed that admission year, advanced age, a higher APACHE IV score and comorbidities were independently associated with a higher risk of hospital mortality.

This study did not show a difference in the risk of hospital mortality between the time to first surgery and appropriateness of antibiotic use groups in the multivariable model. While it may seem common sense that targeting early antimicrobial administration is essential, conclusive high-quality evidence on the exact time threshold for antimicrobial [40] administration in sepsis remains unsettled [41]. A possible reason that this was not shown in this study was because we considered a more conservative approach by classifying unknown with inappropriate initial antibiotics in the multivariate analysis. Similarly, although early surgery and source control is recommended by guidelines, there is conflicting evidence from individual studies on whether early surgery can improve survival in NF [21, 42]. There are a few reasons why our study could not demonstrate benefits of early surgery. First, for patients with sepsis or septic shock, supportive care and resuscitation play a significant role in survival apart from treatment of underlying infection. This is demonstrated by the large adjusted RR of vasopressor therapy, dialysis and APACHE IV score in the multivariable model. Second, even though we used operation booking category as a surrogate to account for preoperative severity, residual bias from other preoperative characteristics may have confounded the effects of surgery time on outcomes. Third, we excluded patients who had surgery only after 24 h of hospital admission. This may have made it harder to demonstrate the benefits of very early surgery (< 6 h) as all patients received surgery within same day of hospital admission [19].

Nonetheless, Nawijn and colleagues (2020) showed a significant reduction in mortality risk associated with surgery within 6 and 12 h after presentation compared to delayed surgery in a systematic review of observational studies [9]. In a post-hoc analysis, the addition of our study data to Nawijn et al.’s meta-analysis of 10 studies (*n* = 512) of surgery within 6 h [9] still gave rise to a significant mortality reduction (RR 0.77, 95% CI: 0.64 to 0.93, I^2^ = 0%; Supplemental Fig. 5) despite a large weighting (60%) from the current study in the random-effects meta-analysis. Similarly, the addition of our study data to the meta-analysis of 15 studies (*n* = 669) of surgery within 12 h [9] leads to a significant mortality reduction (RR 0.70, 95% CI: 0.59 to 0.84, I^2^ = 0%; Supplemental Fig. 6) despite a large weighting (51%) from the current study in the random-effects meta-analysis. Overall, the totality of evidence suggests that surgery within 12 h is associated with a lower risk of hospital mortality. Taken together, our data suggests clinical care could be optimized for at least 12% of patients who are not receiving appropriate empirical antimicrobials and 57% or 27% of patients who are not receiving surgery within 6–12 h of hospital admission.

Our study has several strengths. It is one of the largest retrospective longitudinal cohort studies performed on necrotizing soft tissue infections, involving 15 general ICUs across the whole city of Hong Kong, spanning over 11 years. We standardized the comorbidity categories using the Charlson Comorbidity Index [24]. Our study evaluated the sensitivity patterns of isolated organisms in contrast to many previous studies [9, 20–22] that rarely assessed this. We also examined two key aspects of management — type of surgeries performed and appropriateness of antibiotics used.

On the other hand, there are several limitations. First, as the study lacked information regarding the timing of symptom onset and presentation, the impact of delayed presentation on outcome is uncertain as we only assessed the significance of time interval between hospital admission to surgery on survival. Second, we did not examine the diagnostic process, which is notoriously difficult due to the wide spectrum of presentations. A commonly used predictive scoring system, such as the Laboratory Risk Indicator for Necrotizing Fasciitis (LRINEC) [43], has not been comprehensively externally validated and calibrated. A recent diagnostic test accuracy systematic review of the performance of LRINEC for identifying NF in the extremities showed only fair accuracy [44]. Further studies are needed to validate a robust predictive model for the timely diagnosis of NF, which is crucial in the initial management of such patients. Third, as treatment was not protocolized, the diversity of clinical practice across both different hospitals and time may imposed substantial bias and confounding in this long retrospective multicenter study. Fourth, all-cause mortality at hospital discharge was used and no data on limitation or withdrawal of life sustaining therapy was available.

## Conclusion

Our large retrospective cohort study over 11 years demonstrated a high mortality rate among patients with community-acquired limb NF. Monomicrobial infection constituted most cases, with *S. pyogenes* being the most common causative organism, although marine-related bacteria are also prevalent in our region. Among patients operated within 24 h, very early surgery within 6–12 h was not associated with survival. The admission year, advanced age, high APACHE IV score, comorbidities, RRT, vasopressor use, and type of surgery were significant predictors of hospital mortality.

### Electronic supplementary material

Below is the link to the electronic supplementary material.

Supplementary Material 1

## Data Availability

All data generated or analysed during this study are included in this published article [and its supplementary information files].

## Abbreviations

APACHE IV: Acute Physiology and Chronic Health Evaluation IV
AUROC: area under the receiver operating characteristic curve
CDARS: Clinical Data Analysis and Reporting System
CI: confidence interval
DAG: directed acyclic graph
GAS: group A Streptococcus
ICD-9-CM: International Classification of Diseases, Ninth Revision, Clinical Modification
ICU: intensive care unit
MRSA: Methicillin-resistant Staphylococcus aureus
NF: Necrotizing fasciitis
RR: relative risk
RRT: renal replacement therapy
SMR: standardized mortality ratio
