# Supplementary material for: Association between time to surgery and hospital mortality in patients with community-acquired limb necrotizing fasciitis: an 11-year multicenter retrospective cohort analysis
Source: BMC Infect Dis. 2024 Jun 23;24:624. doi: 10.1186/s12879-024-09501-y (PMC11194942; doi:10.1186/s12879-024-09501-y)
Supplement: Supplementary file 1 — Supplementary Material 1 [file 12879_2024_9501_MOESM1_ESM.pdf]

Supplemental Material

Association between time to surgery and hospital mortality in patients with community-acquired limb necrotizing fasciitis: an 11-year multicenter retrospective cohort analysis

Tables

Supplemental Table 1. Number of surgeries by initial type of surgery

Supplemental Table 2. Microorganisms in patients with inappropriate antibiotics

Supplemental Table 3. Antimicrobial resistance rates of all patients (N = 495)

Supplemental Table 4. Association between time to surgery (<12 hours) and risk of hospital mortality (unadjusted and adjusted relative risk) after taking hospital intensive care unit clustering effect into account in the model

Figures

Supplemental Fig. 1. Directed acyclic graph (DAG) model

Supplemental Fig. 2. Frequency (%) of time to surgery (hours)

Supplemental Fig. 3. Calibration belt for multivariable model examining the association between time to surgery within 6 hours and hospital mortality

Supplemental Fig. 4. Calibration belt for multivariable model examining the association between time to surgery within 12 hours and hospital mortality

Supplemental Fig. 5. Updated forest plot of time of surgery (≤6 hours) on risk of hospital mortality with the inclusion the current study

Supplemental Fig. 6. Updated forest plot of time of surgery (≤12 hours) on risk of hospital mortality with the inclusion the current study

Supplemental Table 1 Number of surgeries by initial type of surgery

Type of Surgery	Median Number of Surgeries (IQR)
Debridement	3 (2-5)
Amputation	2 (1-2)
Debridement then Amputation	3 (2-5)

Supplemental Table 2. Microorganisms in patients with inappropriate antibiotics

**Microorganism**	**Count (%)**<br>**n = 61**
Acinetobacter	2 (3.3)
Aeromonas	5 (8.2)
Arcanobacterium haemolyticum	1 (1.6)
Aspergillus	1 (1.6)
Candida	6 (9.8)
Coagulase negative *Staphylococci species*	8 (13.1)
Escherichia coli	3 (4.9)
Escherichia coli (ESBL)	3 (4.9)
Enterobacter	2 (3.3)
Methicillin-resistant *Staphylococcus aureus*	10 (16.4)
Mycobacterium tuberculosis	1 (1.6)
Proteus	1 (1.6)
Proteus (ESBL)	1 (1.6)
Pseudomonas	2 (3.3)
Scopulariopsis species	1 (1.6)
Serratia	2 (3.3)
Streptococcus pyogenes	4 (6.6)
Streptococcus pyogenes (Clindamycin Resistant)	6 (9.8)
Vibrio	2 (3.3)

This table shows the causative organism in the 61 patients with limb necrotizing fasciitis who were not given appropriate antibiotics within 24 hours of hospitalization. Patients who were not given any antibiotics within 24 hours were included in this group. In patients with polymicrobial infections, the organism that was not covered by the antibiotic regimen was regarded as the causative organism for inappropriate antibiotic therapy.

Supplemental Table 3. Antimicrobial resistance rates of all patients (N = 495)

**Organisms**	**n (%)**
ESBL	7 (1.4)
MRSA	11 (2.2)
CRE	0 (0)
CRAB	0 (0)
VRE	0 (0)

CRAB, carbapenem-resistant *Acinetobacter baumannii*; CRE, Carbapenem-resistant *Enterobacterales*; ESBL, Extended spectrum β-lactamase; MRSA, Methicillin-resistant *Staphylococcus aureus*; VRE, Vancomycin-resistant *Enterococcus*.

Supplemental Table 4. Association between time to surgery (<12 hours) and risk of hospital mortality (unadjusted and adjusted relative risk) after taking hospital intensive care unit clustering effect into account in the model

**Factors**	**Mortality, n (%)**	**Relative Risk (95% CI)**	***p* value**	**Adjusted Relative Risk (95% CI)**	**P value**
Time to Surgery (h)					
≥12	54 (40.9)	1.00		1.00	
<12	127 (35.0)	0.86 (0.62 to 1.19)	0.35	0.87 (0.74 to 1.01)	0.08
Year of admission					
2008	20 (55.6)	1.00		1.00	
2009	14 (45.2)	0.81 (0.47 to 1.41)		0.73 (0.50 to 1.07)	
2010	20 (50.0)	0.90 (0.61 to 1.33)		0.73 (0.48 to 1.10)	
2011	17 (36.2)	0.65 (0.38 to 1.12)		0.60 (0.43 to 0.84)	
2012	16 (32.0)	0.58 (0.32 to 1.04)		0.62 (0.44 to 0.89)	
2013	16 (39.0)	0.70 (0.37 to 1.33)	<0.001	0.52 (0.37 to 0.74)	<0.001
2014	14 (35.0)	0.63 (0.38 to 1.05)		0.63 (0.42 to 0.95)	
2015	6 (13.0)	0.23 (0.13 to 0.44)		0.33 (0.25 to 0.45)	
2016	22 (37.3)	0.67 (0.40 to 1.12)		0.62 (0.41 to 0.95)	
2017	19 (35.2)	0.63 (0.42 to 0.96)		0.70 (0.49 to 1.00)	
2018	17 (33.3)	0.60 (0.42 to 0.86)		0.58 (0.38 to 0.87)	
Age groups (years)					
<45	8 (11.9)	1.00		1.00	
45-64	71 (34.5)	2.89 (1.59 to 5.25)		1.93 (1.21 to 3.07)	
65-74	46 (43.0)	3.60 (1.86 to 6.99)	<0.001	2.16 (1.27 to 3.68)	<0.01
≥75	56 (48.7)	4.08 (2.00 to 8.32)		2.53 (1.48 to 4.34)	
APACHE IV groups					
<60	5 (4.9)	1.00		1.00	
60-89	32 (17.8)	3.63 (2.06 to 6.37)		2.27 (1.27 to 4.10)	
90-119	58 (50.0)	10.2 (5.52 to 18.84)	<0.001	4.84 (2.56 to 9.13)	<0.001
≥120	86 (88.7)	18.09 (11.41 to 28.67)		8.00 (4.89 to 13.09)	
Charlson Comorbidity					
0	63 (23.1)	1.00		1.00	
1	15 (39.5)	1.71 (1.03 to 2.83)		1.32 (0.96 to 1.82)	
2	34 (44.7)	1.94 (1.36 to 2.76)	<0.001	1.38 (1.11 to 1.72)	<0.001
≥3	69 (63.9)	2.77 (2.28 to 3.36)		1.70 (1.47 to 1.96)	

Type of infection					
Monomicrobial	140 (39.8)	1.00		1.00	
Polymicrobial	32 (32.0)	0.81 (0.68 to 0.96)	0.01	0.97 (0.76 to 1.23)	0.36
No growth	9 (20.9)	0.54 (0.30 to 0.97)		0.68 (0.39 to 1.17)	
Bacteraemia					
No	85 (26.2)	1.00		1.00	
Yes	96 (56.5)	2.16 (1.76 to 2.65)	<0.001	1.12 (0.89 to 1.40)	0.34
Inappropriate antibiotics					
No or unknown	157 (36.2)	1.00		1.00	
Yes	24 (39.3)	1.09 (0.76 to 1.55)	0.64	1.08 (0.79 to 1.46)	0.64
Mechanical ventilation					
No	3 (5.5)	1.00		1.00	
Yes	178 (40.5)	7.42 (2.97 to 18.53)	<0.001	2.66 (0.91 to 7.78)	0.07
Renal replacement therapy					
No	93 (25.2)	1.00		1.00	
Yes	88 (69.8)	2.77 (2.15 to 3.57)	<0.001	1.33 (1.10 to 1.61)	<0.01
Vasopressor					
No	89 (31.0)	1.00		1.00	
Yes	92 (44.2)	1.43 (1.17 to 1.73)	<0.01	1.26 (1.01 to 1.58)	0.04
Type of surgery					
Debridement only	75 (27.1)	1.00		1.00	
Amputation only	73 (58.9)	2.17 (1.77 to 2.67)	<0.001	1.16 (1.01 to 1.33)	<0.001
Debridement then amputation	33 (35.1)	1.30 (1.00 to 1.69)		0.88 (0.68 to 1.15)	
Urgency of surgery					
Semi-urgent	24 (28.9)	1.00		1.00	
Urgent	150 (37.5)	1.30 (0.94 to 1.79)	<0.01	1.00 (0.75 to 1.35)	1.00
Unknown	7 (58.3)	2.02 (1.33 to 3.06)		1.00 (0.69 to 1.45)	

APACHE IV, Acute Physiology And Chronic Health Evaluation IV

Supplemental Fig. 1. Directed acyclic graph (DAG) model

Assumptions made in the time to surgery (exposure) and hospital mortality (outcome) relationship to identify the set of covariates for adjustment. Blue circle represents ancestor of outcome. Red circle represents ancestor of exposure and outcome. All red arrows lie on open biasing paths. All green arrows lie on open causal paths. Bold arrows indicate no corresponding indirect path (no causal effect between variables exists if arrow is removed). Thin arrows indicate that there is another indirect pathway between variables.

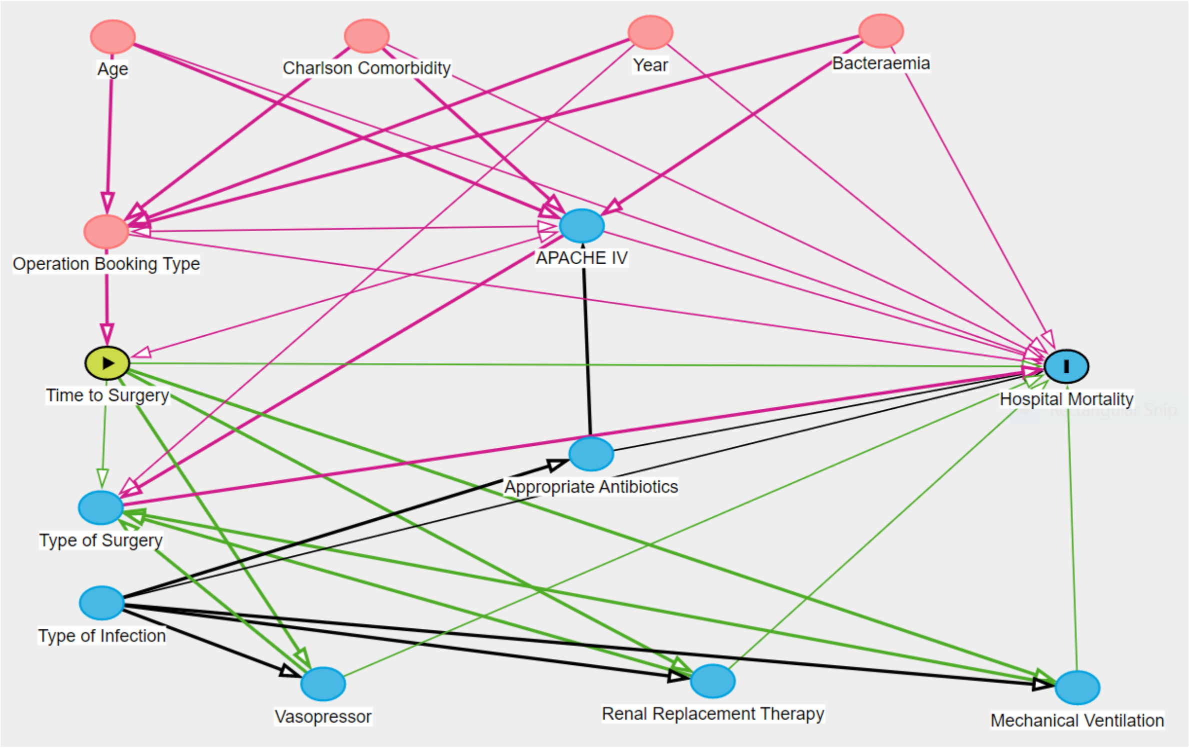

Supplemental Fig. 2. Frequency (%) of time to surgery (hours)

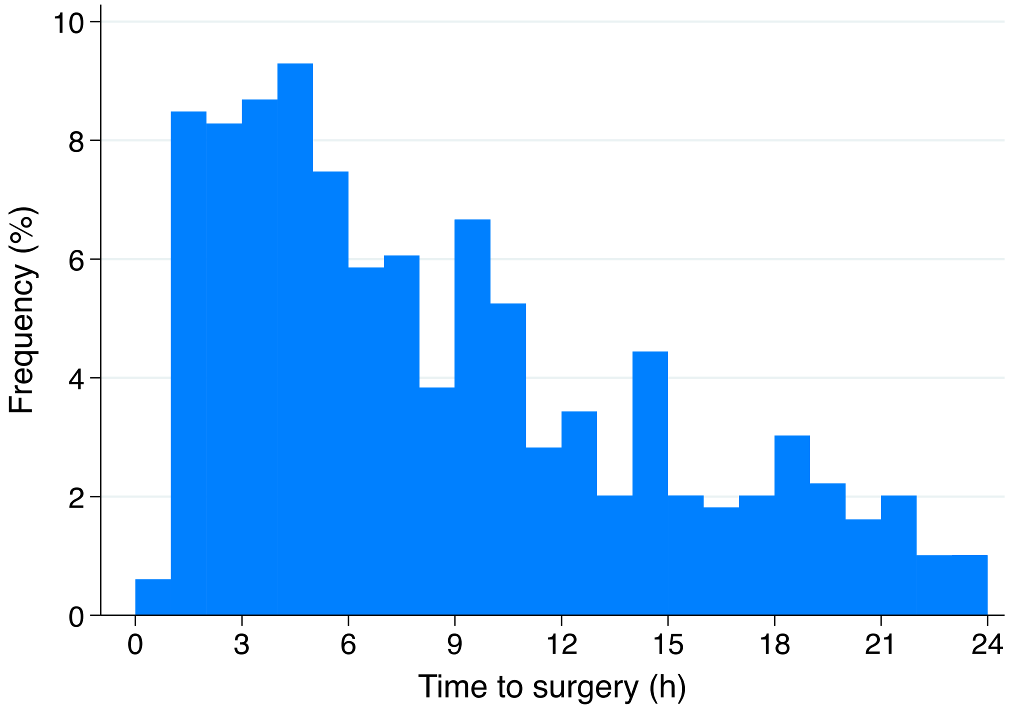

Supplemental Fig. 3. Calibration belt for multivariable model examining the association between time to surgery within 6 hours and hospital mortality

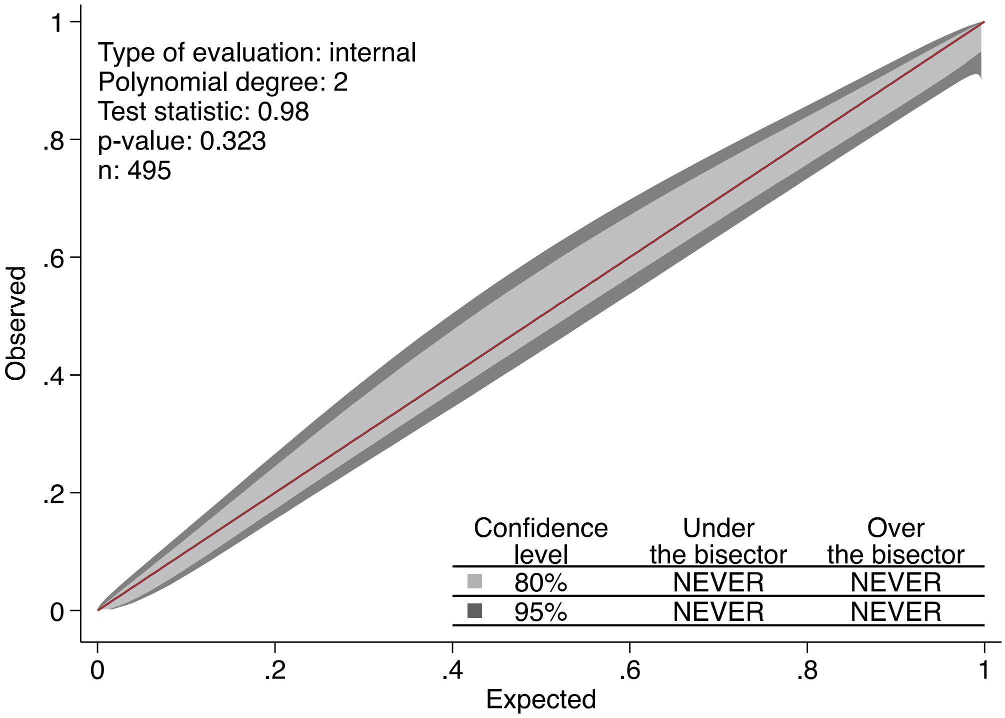

Supplemental Fig. 4. Calibration belt for multivariable model examining the association between time to surgery within 12 hours and hospital mortality

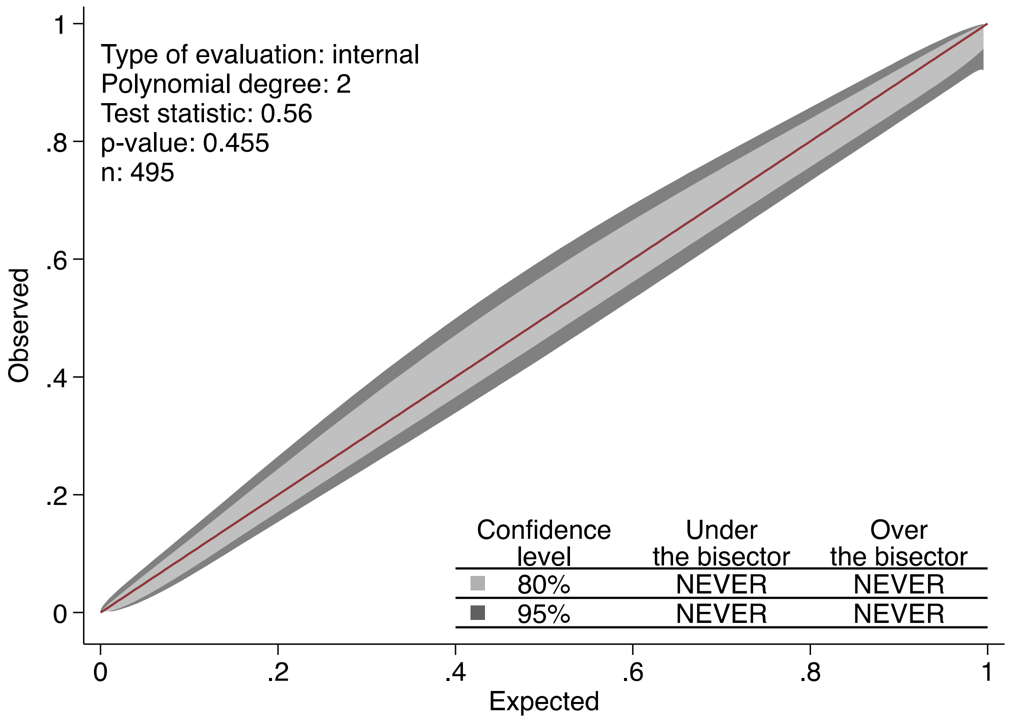

Supplemental Fig. 5. Updated forest plot of time of surgery (<6 hours) on risk of hospital mortality with the inclusion the current study

Study	Treatment Yes	Treatment No	Control Yes	Control No	Risk Ratio with 95% CI	Weight (%)
Boyer 2009	8	4	16	5	0.88 [0.55, 1.39]	15.80
Chao 2013	4	23	31	63	0.45 [0.17, 1.16]	3.81
Corona 2016	2	7	4	7	0.61 [0.14, 2.61]	1.63
Hadeed 2016	3	37	8	39	0.44 [0.13, 1.55]	2.17
Huang 2008	0	11	3	2	0.07 [0.00, 1.17]	0.44
Kaiser 1981	3	10	5	2	0.32 [0.11, 0.97]	2.85
Latifi 2018	4	30	15	66	0.64 [0.23, 1.78]	3.25
Nawijn 2019a	2	18	1	4	0.50 [0.06, 4.47]	0.71
Nawijn 2019b	8	22	12	15	0.60 [0.29, 1.24]	6.48
Tsai 2015	5	6	3	4	1.06 [0.36, 3.10]	2.98
Current study	71	141	110	173	0.86 [0.68, 1.09]	59.87
**Overall**					0.77 [0.64, 0.93]	

Heterogeneity: $\tau^2 = 0.00$, $I^2 = 0.00\%$, $H^2 = 1.00$

Test of $\theta_i = \theta_j$: $Q(10) = 9.49$, $p = 0.49$

Test of $\theta = 0$: $z = -2.77$, $p = 0.01$

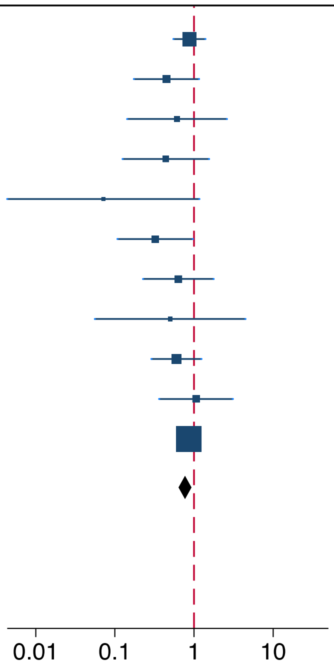

Random-effects DerSimonian–Laird model

Supplemental Fig. 6. Updated forest plot of time of surgery (<12 hours) on risk of hospital mortality with the inclusion the current study

Study	Treatment Yes	Treatment No	Control Yes	Control No	Risk Ratio with 95% CI	Weight (%)
Balci 2009	0	6	2	3	0.17 [0.01, 2.92]	0.39
Boyer 2009	9	7	15	2	0.64 [0.40, 1.02]	14.61
Chao 2013	8	45	27	41	0.38 [0.19, 0.77]	6.42
Corona 2016	3	11	3	3	0.43 [0.12, 1.55]	1.92
Huang 2008	2	11	1	2	0.46 [0.06, 3.57]	0.76
Kaiser 1981	4	11	4	1	0.33 [0.13, 0.86]	3.54
Kobayashi 2011	1	21	7	18	0.16 [0.02, 1.22]	0.78
Lee 2014	10	57	8	25	0.62 [0.27, 1.41]	4.59
Lille 1996	1	16	3	9	0.24 [0.03, 2.00]	0.69
Nawijn 2019a	3	21	0	2	0.84 [0.06, 12.63]	0.43
Nawijn 2019b	12	30	9	19	0.89 [0.43, 1.83]	6.11
Pakula 2012	5	30	3	16	0.90 [0.24, 3.38]	1.82
Sudarsky 1987	0	7	2	24	0.68 [0.04, 12.67]	0.37
Tsai 2010	12	42	6	11	0.63 [0.28, 1.42]	4.78
Tsai 2015	7	9	1	1	0.88 [0.20, 3.89]	1.42
Current study	127	236	54	78	0.86 [0.67, 1.10]	51.36
**Overall**					0.70 [0.59, 0.84]	

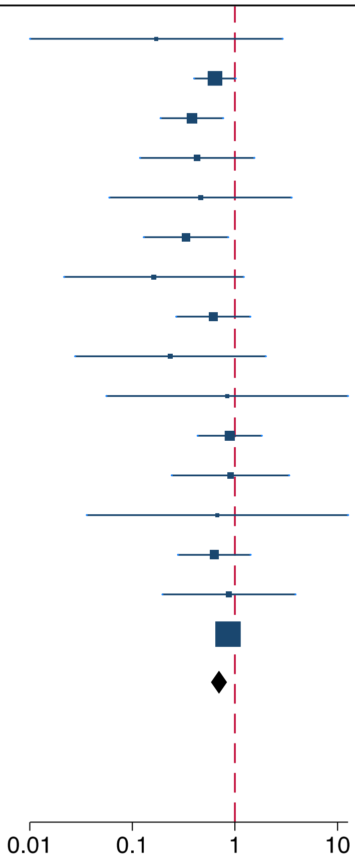

Heterogeneity: $\tau^2 = 0.00$, $I^2 = 0.00\%$, $H^2 = 1.00$

Test of $\theta_i = \theta_j$: Q(15) = 13.43, p = 0.57

Test of $\theta = 0$: z = -3.94, p = 0.00

Random-effects DerSimonian–Laird model
